# Tumor acidosis enhances cytotoxic effects and autophagy inhibition by salinomycin on cancer cell lines and cancer stem cells

**DOI:** 10.18632/oncotarget.9601

**Published:** 2016-05-27

**Authors:** Paola Pellegrini, Matheus Dyczynski, Francesca Vittoria Sbrana, Maria Karlgren, Maria Buoncervello, Maria Hägg-Olofsson, Ran Ma, Johan Hartman, Svetlana Bajalica-Lagercrantz, Dan Grander, Pedram Kharaziha, Angelo De Milito

**Affiliations:** ^1^ Department of Oncology-Pathology, Cancer Center Karolinska, Karolinska Institute, Stockholm, Sweden; ^2^ Istituto Ortopedico Rizzoli, Bologna, Italy; ^3^ Department of Pharmacy and Uppsala University Drug Optimization and Pharmaceutical Profiling Platform (UDOPP) - Science for Life Laboratory, Department of Pharmacy, Uppsala Biomedical Center, Uppsala University, Sweden; ^4^ Istituto Superiore di Sanità, Rome, Italy

**Keywords:** Autophagy, chloroquine, tumor acidosis, cancer therapy, pH

## Abstract

Sustained autophagy contributes to the metabolic adaptation of cancer cells to hypoxic and acidic microenvironments. Since cells in such environments are resistant to conventional cytotoxic drugs, inhibition of autophagy represents a promising therapeutic strategy in clinical oncology. We previously reported that the efficacy of hydroxychloroquine (HCQ), an autophagy inhibitor under clinical investigation is strongly impaired in acidic tumor environments, due to poor uptake of the drug, a phenomenon widely associated with drug resistance towards many weak bases. In this study we identified salinomycin (SAL) as a potent inhibitor of autophagy and cytotoxic agent effective on several cancer cell lines under conditions of transient and chronic acidosis. Since SAL has been reported to specifically target cancer-stem cells (CSC), we used an established model of breast CSC and CSC derived from breast cancer patients to examine whether this specificity may be associated with autophagy inhibition. We indeed found that CSC-like cells are more sensitive to autophagy inhibition compared to cells not expressing CSC markers. We also report that the ability of SAL to inhibit mammosphere formation from CSC-like cells was dramatically enhanced in acidic conditions. We propose that the development and use of clinically suitable SAL derivatives may result in improved autophagy inhibition in cancer cells and CSC in the acidic tumor microenvironment and lead to clinical benefits.

## INTRODUCTION

Malignant cells develop under the selective pressure of metabolic stress conditions including chronic and intermittent hypoxia and limited nutrients availability [1–3]. Metabolic adaptation to such environment allows rapid and continuous growth of cancer cells, leading to developing of abnormal blood vessels, which arrange irregularly and are structurally heterogeneous [4]. The vascular endothelium in tumors is defective and leaky [5] and defective tissue perfusion coupled with high metabolic rates contribute to extracellular acidosis in the tumor microenvironment [6, 7]. It has been shown in different animal models that the tumor extracellular pH (pHe) ranges between 5.9 and 7.2 [8–10], with average tumor pHe often reported ~6.5 [11–13]. Tumor acidosis represents an important selective force affecting cancer progression and therapeutic resistance [1, 14–18]. Indeed, efforts and strategies to counteract tumor acidosis have shown promising results in different preclinical models [3, 13, 18–20].

Macroautophagy (autophagy) is an evolutionary conserved process that contributes to cellular homeostasis by degrading damaged or redundant organelles, and misfolded proteins [21]. The process begins with the formation of a double membrane structure, which engulfs parts of the cytoplasm creating a double membrane vesicle, which fuses with lysosome (autolysosome). Lysosomal enzymes active in the acidic environment of autolysosomes degrade the sequestered cargo and provide hydrocarbons, fatty acids, amino acids and nucleotides to cells. Autophagy has a context-dependent role in initiation, development and progression of cancer [22, 23]. Autophagy suppresses malignant transformation by different mechanisms such as protection from genomic instability through elimination of dysfunctional mitochondria and decreased reactive oxygen species (ROS) production, degradation of onco-proteins, induction of senescence and cell death following oncogene activation, anti-inflammatory function and helping the immune system to eliminate the malignant cells [24]. After establishment of a neoplastic lesion, autophagy is believed to promote and sustain tumorigenesis and to mediate resistance to different forms of anticancer therapy [25]. In fact, proficient autophagy contributes to metabolic reprogramming and adaptation to different metabolic stresses, including hypoxia, acidosis and nutrient deprivation. About seventy clinical trials are registered in clinicaltrials.gov testing the use of HCQ or chloroquine (CQ) as autophagy inhibitors in combination anticancer therapies [26] but the use of CQ derivatives for this purpose may require further investigation. In fact, results from the first phase I/II trials support autophagy inhibition as a promising therapeutic strategy but also indicate limitation in the efficacy of HCQ to inhibit autophagy at the tumor site [27–32], underlining the need to identify better and more specific autophagy inhibitors [33]. Recently, others and we reported that autophagy contributes to the survival and adaptation of cancer cells to an acidic environment [34, 35]. However, CQ and HCQ fail to inhibit autophagy in acidic conditions because of a reduction in cellular uptake of the drugs [36]. Interestingly, the new CQ-derivative Lys-01, which displays lower pKa values, has a better activity both as cytotoxic agent and autophagy inhibitor in acidic conditions [36, 37].

CSC represent a subpopulation of cells with tumor initiating ability and they are believed to contribute to resistance to chemotherapy, metastatic spread and disease relapse [38]. It has also been suggested that tumor development is characterized by a dynamic transition between CSC and differentiated tumor cells and that both these subpopulations should be considered as target for therapy [39]. Gupta et al. reported that SAL, a polyether antibiotic widely used in veterinary medicine is a potent agent able to selectively target breast CSC and to inhibit mammary tumor growth *in vivo* [40]. It has been reported that autophagy promotes maintenance of breast CSC and tumorigenicity [41, 42] and that SAL can inhibit autophagy and lysosomal proteolytic activity in both breast CSC and cancer cells [43]. SAL has been described as a potassium ionophore inhibiting Wnt signaling and interfering with the proton gradient within lysosomes [44], although no effect on lysosomal pH have been reported in SAL-treated breast cancer cells [43].

In this study we analysed the pH-dependent cytotoxic and autophagy inhibiting activities of SAL towards cancer cell lines and CSC. We found that SAL is a potent inhibitor of the autophagic flux and cytotoxic agent showing increased efficacy towards cancer cells under low pH conditions.

## RESULTS

### Salinomycin is a potent autophagy inhibitor in acidic conditions

**Figure 1:**
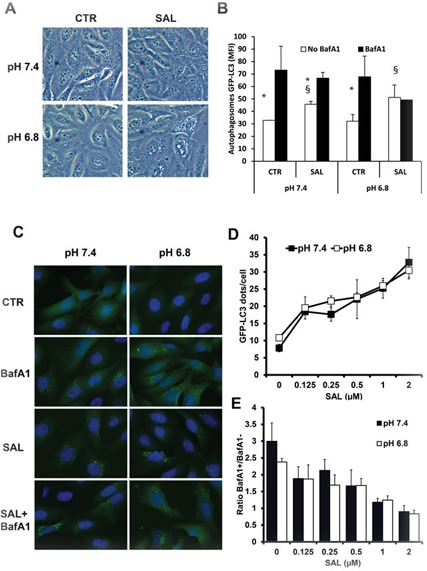
Effects of SAL on accumulation of GFP-LC3+ vesicles in HOS cells at pH 7.4 and 6.8 **A.** Phase contrast microscopy pictures of HOS-GFP-LC3 cells cultured with medium buffered at pH 7.4 or pH 6.8 overnight and treated with SAL (2 μM) for 6 hours. **B.** In the same settings as in A, BafA1 (50 nM) was added for 2 hours before flow cytometric analysis of saponin-permeabilised cells to quantify autophagosomes-associated GFP-LC3 (data are from three independent experiments run in duplicate samples). Differences between MFI in the presence or absence of BafA1 is indicated by *, showing a *P* < 0.05. Differences between MFI in the presence or absence of SAL is indicated by §, showing a *P* < 0.05. **C.** Fluorescence microscopy images of GFP-LC3-HOS cells under different treatments showing the presence of GFP-LC3+ vesicles. **D.** Quantitative analysis of the dose-dependent activity of SAL on the number of GFP-LC3+ vesicles run in triplicates. **E.** The ratio between the number of GFP-LC3+ vesicles in presence and absence of BafA1 is shown. Ratio of 1 indicates no additive effects of BafA1 on the SAL-mediated inhibition of the autophagic flux.

We recently showed that the clinically used autophagy inhibitors CQ and HCQ are not effective in blocking autophagy in the acidic environment of human tumors [36]. This effect was associated with a complete lack of cytotoxicity in acidic conditions in several cancer cell lines. In search of new autophagy inhibitors active also in acidic conditions we focused on SAL, an acidic ionophore compound used as anticoccidiosis in veterinary medicine. SAL was reported to induce cell death *via* autophagy upregulation in some experimental models [45, 46]. However, it was recently reported that 2 μM SAL inhibits the autophagic flux in breast and hepatocellular carcinomas [43, 47]. In order to establish the activity of SAL on autophagic flux, we started our investigation by using HOS cells stably transfected with a GFP-LC3 vector, which allows the analysis of the autophagic flux by flow cytometry by monitoring the accumulation of GFP-LC3-positive autophagosomes in the presence of lysosomal inhibitors [48]. BafA1 acts as inhibitor of the V-ATPase and raises lysosomal pH, thus inhibiting autolysosomes formation and leading to accumulation of GFP-LC3-positive autophagosomes. The autophagic flux here represents the ratio of GFP-LC3 fluorescence between presence and absence of saturating concentration of Bafilomycin A1 (BafA1). First, we observed that HOS-GFP-LC3 cells treated with 2 μM SAL for 6 hours accumulate a large number of intracellular vacuoles, with cells cultured at pH 6.8 showing an increased vacuolization with respect to cells kept at pH 7.4 (Figure 1A). As expected, autophagosomes-associated LC3-GFP fluorescence was increased in control cells treated with BafA1 at both pH conditions, indicating the presence of proficient autophagy in both pH conditions (Figure 1B), with autophagic flux being 2.2±0.23 and 2.2±0.36, respectively at pH 7.4 and 6.8. A significant increase in GFP-LC3 fluorescence was observed also in cells treated only with SAL in both pH conditions. The combined treatment with BafA1 showed only a minor increase in cells at pH 7.4, indicating that SAL reduces the autophagic flux without blocking it (1.5±0.1). Conversely, in cells kept at pH 6.8 and treated with SAL the GFP-LC3 signal intensity was similar in presence or absence of BafA1, suggesting that in HOS cells in acidic conditions SAL totally blocks the autophagic flux (1±0.1, Figure 1B). To further test the dose-dependent effects of SAL in these cells we used high-content fluorescence microscopy to quantify the number of GFP-LC3-positive vesicles in cells treated with different doses of SAL in absence or presence of BafA1. The data show that SAL induces a dose-dependent accumulation of GFP-LC3-positive vesicles (Figure 1C-1D) and that at 1-2 μM SAL the number of GFP-LC3-positive vesicles in BafA1 treated and untreated cells is similar, thus suggesting that SAL inhibits autophagic flux (Figure 1E).

**Figure 2:**
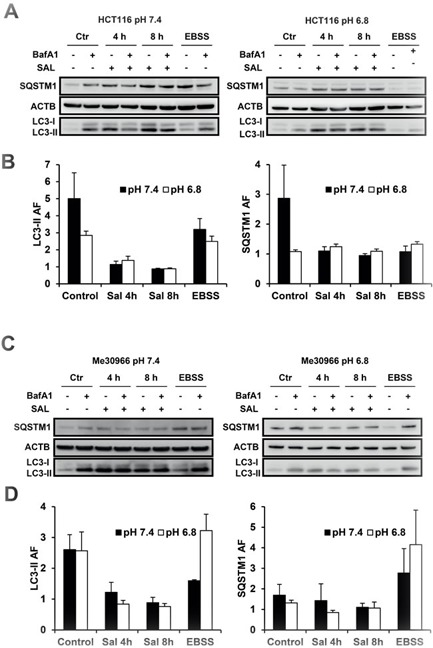
Effects of SAL on autophagic flux in cancer cell lines at pH 7.4 and 6.8 Parental and low pH-adapted HCT116 **A.** and Me30966 **C.** cells were exposed to SAL (2 μM) for 4-8 hours, with or without BafA1 (100 nM) during the last 2 hours. WB analysis of LC3-II and SQSTM1 was performed to measure the autophagic flux. EBSS-treated cells were used as controls. Data in panels A and C are representative of four independent experiments. Quantification of LC3-II and SQSTM1 are shown for HCT116 **B.** and Me30966 **D.** cells with data expressed as mean and SE.

In order to properly demonstrate the pH-dependent activity of SAL, we extended our analysis to different cell lines representing different tumor hystotypes and in conditions of both transient and chronic acidosis. First, we used HCT116 (colon carcinoma) and Me30966 (melanoma) cell lines from which we developed sub-lines chronically adapted to grow at pH 6.8 [36]. As indicated above, BafA1 as a lysosomal inhibitor blocks the degradation and turnover of LC3-II and other substrates such as SQSTM1. The autophagic flux was then defined as the ratio between the normalised LC3-II levels in presence and absence of BafA1 at saturating concentrations (100 nM) (LC3-II AF). In these settings, a ratio of 1 indicates total blockage of autophagic flux. As observed in the HOS-GFP-LC3 cells, SAL induced a dose-dependent inhibition of the turnover of both LC3-II and SQSTM1 in all cell lines tested, with best activity detected at 2 μM (Figure S1A-B). As shown in Figure 2A and 2C, SAL treatment induced a strong time-dependent increase in LC3-II expression in both parental and pH 6.8-adapted HCT116 and Me30966 cells. In all cell lines, autophagic flux was strongly inhibited already after 4 hours treatment. Despite some variability likely dependent on different substrate-specific kinetics, the turnover of both LC3-II and to a lesser degree SQSTM1 was reduced in all cell lines independently of the pH culture conditions (Figure 2B and 2D).

**Figure 3:**
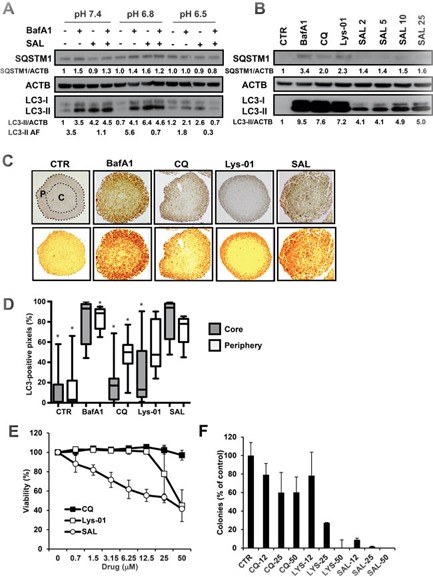
Effects of SAL on HCT116 cells at pH 6.5 and in multicellular spheroids **A.** HCT116 cells were exposed to medium at pH 7.4, pH 6.8 and pH 6.5 overnight. SAL (2 μM) was added to cells for 6 hours, with or without BafA1 (100 nM) during the last 2 hours. The picture shows one representative WB analysis of three independent experiments. **B.** MCS derived from HCT116 cells were treated for 24 hours with different autophagy inhibitors (50 nM BafA1, 50 μM CQ and Lys-01) and SAL at different concentrations (μM). WB analysis on cell lysates was performed to analyse the expression of LC3-II and a representative WB from three different experiments is shown. **C.**-**D.** HCT116 MCS (*n* = 24) were treated with different autophagy inhibitors for 24 hours and analysed by IHC to detect the distribution of the augmented LC3 expression. LC3 positive pixel analysis was performed in the peripheral (P) and core (C) regions of the MCS as indicated for one representative untreated MCS. Data are shown as box plots, normalised and expressed as percentage of the total number of pixels in the specific area. The * indicates a *P* < 0.05 for differences between SAL and all treatment groups, calculated by the Mann-Whitney test. **E.** HCT116 MCS were treated for 3 days with different concentrations of CQ, Lys-01 and SAL. Cell viability was determined with the acid phosphatase assay. **F.** HCT116 MCS were treated for 3 days with different concentrations of CQ, Lys-01 and SAL. Single cell suspensions were obtained from MCS and clonogenic survival assay was performed. Experiments in E-F were repeated three times and means and SD are shown.

The data shown so far suggest that SAL efficiently inhibits the autophagic flux in cancer cells also at pH 6.8. However, tumor tissues may be characterised by regions with even lower pH and the average pH in tumors is reported to be around 6.5 [10, 12, 16, 49]. We have reported that CQ failed to inhibit autophagy at pH 6.8 while the CQ dimer Lys-01 had still a detectable activity at pH 6.8. However, when tested at pH 6.5, Lys-01 did not induce LC3-II increase in HCT116 cells, suggesting that its activity is also inhibited at very acidic conditions (Figure S1C). Thus, we tested the activity of SAL on the autophagic flux in cells after exposing them to medium buffered at pH as low as 6.5. We exposed HCT116 cells to media buffered at different pH overnight and added 2 μM SAL for 8 hours, with BafA1 added during the last 2 hours incubation. We observed that SAL maintained a strong autophagy blocking activity even at pH 6.5 as shown by LC3-II and SQSTM1 expression (Figure 3A) and a similar observation was made using Me30966 cells cultured at pH 6.5 (Figure S1D).

### The activity of SAL is detectable in the acidic core of multicellular spheroids

To gain further insight into the capacity of SAL to block autophagy in a more complex environment we took advantage of the multicellular spheroid (MCS) culture system, where cells grow in a 3D structure with a central core characterised by acidic pH in the range 6.6-6.8 [50]. MCS from HCT116 cells were treated with CQ, Lys-01 and SAL and the accumulation of LC3-II was analysed. First, we observed by Western Blot (WB) that SAL induced a dose-dependent accumulation of LC3-II and SQSTM1 after 24 hours treatment (Figure 3B). The accumulation of LC3-II was even higher in MCS treated with Lys-01 (50 μM), BafA1 (50 nM) and CQ (50 μM).

Compounds blocking the autophagic turnover of LC3-II are expected to induce an increase in total LC3 positivity in tissues due to lack of LC3-II degradation. Interestingly, when analyzing the distribution pattern of LC3 signal intensity in relation to MCS areas by immunohistochemistry (IHC), we observed that MCS treated with CQ and Lys-01 showed a strong LC3 signal localised in the outer peripheral layer but almost completely absent in the MCS core, suggesting a failure to block autophagy in the acidic/hypoxic core of the MCS (Figure 3C). Conversely, low concentrations of SAL (2 μM) were able to increase LC3 intensity throughout the whole MCS area, similarly to the effects observed after treatment with BafA1 (Figure 3C). A quantitative image analysis of LC3 positive pixels normalised to the total number of pixels in each area showed that CQ and Lys-01 mostly induced accumulation of LC3 in the peripheral layer of MCS while BafA1 and SAL induced a significantly higher increase in LC3 positive pixels both in the peripheral region and in the core region of MCS with respect to CQ and Lys-01 (Figure 3C-3D).

In line with a more effective autophagy blocking activity in this 3D culture model, SAL showed a much stronger cytotoxic effect on MCS as compared to CQ and Lys-01 as shown by viability assay (Figure 3E). Interestingly, clonogenic cell survival assay showed that while 12 μM CQ or Lys-01 still allowed about 80% of treated cells to form colonies, the same concentration of SAL completely inhibited the capacity of cells to survive treatment (Figure 3F).

### SAL blocks the autophagic flux in cancer stem cells

SAL was identified as a breast CSC-specific cytotoxic agent using a model of immortalised and transformed breast carcinoma cells (HMLER) carrying CSC properties [40]. Using the same model, Yue and colleagues reported that SAL selectively targets breast CSC through inhibition of autophagy [43]. HMLER cells are composed of two different cell populations differing in their CSC properties and in CD24 expression (Figure S2A) [51]. CD44+/CD24+ cells have an epithelial phenotype and no ability to make mammospheres while CD44+/CD24low cells have properties of CSC, including capacity to form mammospheres. First, we observed also in these cells that 2 μM SAL blocks the autophagic flux both at pH 7.4 and pH 6.8 as measured by LC3-II in the presence of BafA1 (Figure S2C).

**Figure 4:**
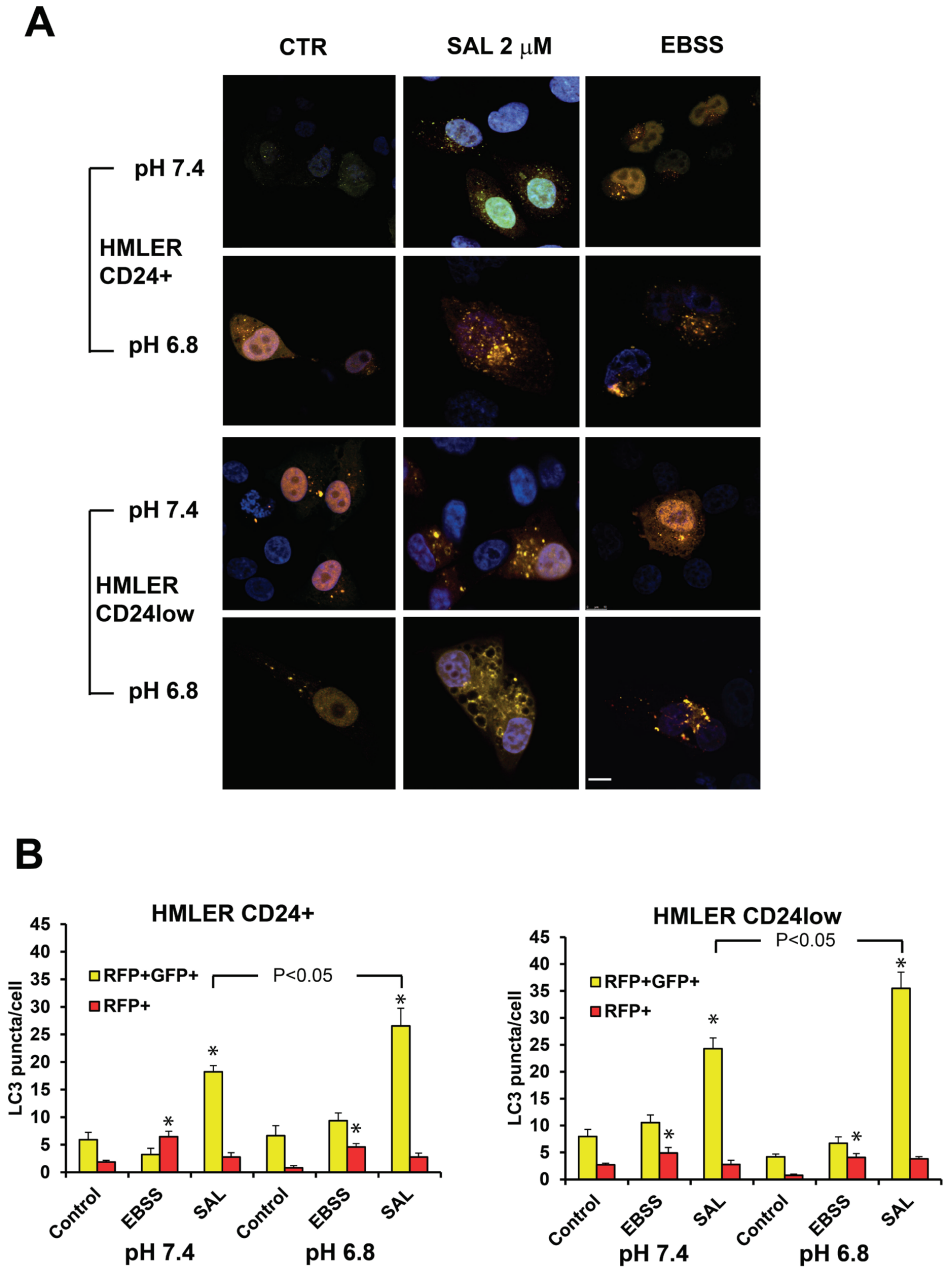
Effects of SAL on autophagy in HMLER cells by fluorescence microscopy **A.** HMLER CD24+ and CD24low cells were transfected with RFP-GFP-LC3 plasmid and then cultured with medium buffered at pH 7.4 or pH 6.8 overnight. SAL (μM) was added at different concentrations for 6 hours and cells treated with EBSS were used as positive control. Cells were fixed and observed with confocal microscopy. **B.** The number of yellow and red dots per cell was analysed and data in the charts are expressed as mean ± SE from at least 15 cells/condition. Scale bar: 10 μm. The * indicates a *P* < 0.05 for differences between treatment groups and controls.

In order to confirm the data on autophagic flux obtained by WB, we transfected HMLER CD24+ and CD24low cells with an LC3 tandem probe in which LC3 is tagged to GFP and RFP. Because the acidic pH of lysosomes quenches the green fluorescence of GFP, this probe helps to identify and count autophagosomes (GFP+RFP+ yellow dots) and autolysosomes (GFP-RFP+ red dots). Cells were exposed overnight to medium buffered at pH 7.4 or 6.8 and treated with SAL (2 μM) or starved with Earle's Balanced Salt Solution (EBSS) for 6 hours (Figure 4A). As expected, EBSS-treated cells showed an increased number of red dots in all conditions examined, indicating that starvation-induced autophagy occurs in all cells (Figure 4B). Treatment with SAL induced the accumulation of yellow dots in all conditions tested as compared to control and such effect was significantly greater in both cell lines cultured at acidic pH (Figure 4B).

**Figure 5:**
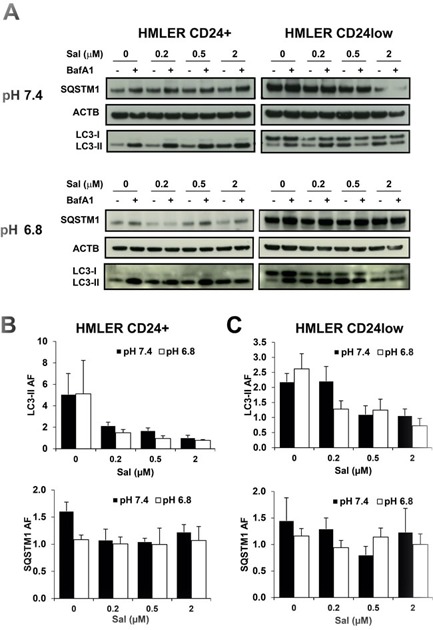
Effects of SAL on autophagy in HMLER cells by western blot **A.** HMLER CD24+ and CD24low cells were plated and cultured with medium buffered at pH 7.4 or pH 6.8 overnight. SAL (μM) was added at different concentrations for 6 hours, with or without BafA1 (100 nM) during the last 2 hours incubation. The picture shows one representative WB analysis of four different experiments. Quantification of LC3-II and SQSTM1 is shown in panel **B.** for CD24+ cells and panel **C.** for CD24low cells and data are expressed as mean and SE from four independent experiments.

To further investigate the sensitivity of HMLER cells to autophagy inhibition we tested the activity of different SAL concentrations at pH 7.4 and pH 6.8. First, we observed that the basal autophagic turnover of LC3-II seems to be higher in CD24+ cells as compared to CD24low cells (Figure 5A). The analysis of the autophagic flux based on LC3-II turnover showed that SAL reduced the autophagic flux in a dose-dependent manner in both cell lines and at both pH 7.4 and pH 6.8 (Figure 5A-5C). Quantification of the autophagic flux calculated based on BafA1-induced accumulation of SQSTM1 supported the data obtained with LC3-II (Figure 5B, 5C).

In conclusion, these data suggest that very low SAL concentrations are able to inhibit the autophagic flux in HMLER cells cultured at acidic pH.

### Acidosis enhances the cytotoxicity of salinomycin against cancer cells

**Figure 6:**
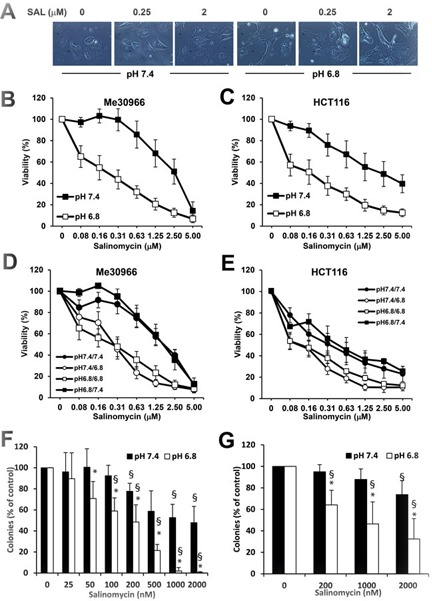
Cytotoxic activity of SAL on parental and acid-adapted HCT116 and Me30966 cells **A.** Phase contrast microscopy pictures of Me30966 cells treated with SAL for 5 hours. **B.**-**C.** Viability assay with acid phosphatase on Me30966, HCT116 and their pH 6.8 adapted sublines after 48 hours treatment with different concentrations of SAL. **D.**-**E.** Parental and low pH-adapted Me30966 (D) and HCT116 (E) cells were plated in their respective medium at pH 7.4 and pH 6.8. The next day the medium was replaced with either medium with the same pH or medium with a different pH. Viability was assessed after 48 hours treatment with different concentrations of SAL. The experiment was repeated five times in triplicate wells and data show means and SD. **F.**-**G.** Clonogenic assay on parental and pH 6.8-adapted HCT116 cells treated with SAL for 8 hours (F) or 48 hours (G) and then left in drug-free medium for 6 days. Data in B-E are obtained from at least three different experiments and expressed as mean ± SD. Differences between groups in F-G were analysed with paired T-test. * indicates a *P* < 0.05 for differences between pH 7.4 and pH 6.8 while § indicates a *P* < 0.05 for differences between SAL-treatment and controls. Non significant differences are not shown.

The inability of CQ to block autophagy in acidic conditions is associated with a complete lack of cytotoxicity in cancer cells under acidic conditions. Therefore, we performed cytotoxic assays to test whether low pH affected the cytotoxic effects of SAL. We used parental HCT116 and Me30966 cells cultured at pH 7.4 and their low pH-adapted sublines cultured at pH 6.8 (HCT116_pH7.4_ and HCT116_pH6.8_) and exposed to increasing SAL concentrations for 48 hours. Microscopic observation of cells indicated a massive vacuolization at pH 6.8 already 6 hours after treatment with SAL, as shown for Me30966 cells in Figure 6A. SAL showed a dramatic and significantly increased cytotoxicity in low pH-adapted cells (Figure 6B-6C), with a 10-fold difference in IC_50_ values (Table 1). These effects were not dependent on the phenotype of acid-adapted cells since parental HCT116 and Me30966 cells exposed to acidic medium only for the duration of the treatment were equally sensitive as their acid-adapted sublines (Figure 6D-6E), suggesting that the increased SAL cytotoxicity in acidic conditions are likely dependent only on the pH of the culture medium. Conversely, the cytotoxic effects of SAL in acid-adapted cells cultured at pH 7.4 were comparable to those observed for the parental cell lines (Figure 6D-6E).

**Table 1:** The IC_50_ (μM) of SAL in HCT116 and Me30966 cells is reported

	HCT116	Me30966
**pH 7.4**	1.63 ± 0.77	2.46 ± 0.62
**pH 6.8 adapted**	0.12 ± 0.06	0.22 ± 0.15
**pH 6.8 transient**	0.13 ± 0.07	0.54 ± 0.19

To analyse the effects of SAL on clonogenic cell survival we used HCT116_pH7.4_ and HCT116_pH6.8_ cells and exposed them to different SAL concentrations for 48 hours, followed by culturing cells in drug-free medium for 6 days. Again, the inhibiting effect of SAL on clonogenic growth was dramatically increased in cells cultured at acidic pH (Figure 6F). In addition, we performed clonogenic survival assay on parental HCT116 cells cultured at pH 7.4 and pH 6.8 in the presence of SAL for 8 hours and then kept in drug-free medium for 6 days. The data indicate that even short exposure to SAL in acidic conditions is effective in reducing clonogenic cell survival while very mild effects were observed in cells treated at pH 7.4 (Figure 6G).

### Acidic conditions improve the ability of SAL to kill cancer stem cells

**Figure 7:**
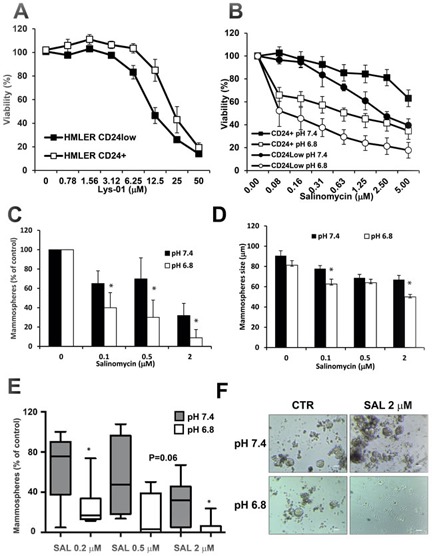
Cytotoxic activity of SAL on breast cancer stem cells **A.** Cell viability of HMLER CD24+ and CD24low cells after treatment with the autophagy inhibitor Lys-01 for 48 hours. **B.** Cell viability of HMLER CD24+ and CD24low cells cultured with medium buffered at different pH and treated with SAL for 48 hours. **C.** CD24low cells were treated with SAL for 48 hours at two pH conditions and viable cells were allowed to form mammospheres for 6 days. Salinomycin reduces the sphere-forming capacity of CD24low cells and the effects are increased when treatment is done in acidic conditions. **D.** Mammospheres size from experiments in panel D is shown. Data in panels C-D are obtained from four different experiments and expressed as mean ± SD. **E.** CSC were isolated from six patients with breast carcinoma. Single cell suspensions obtained from patients-derived mammospheres were cultured in medium buffered at pH 7.4 or 6.8 for 10 days and the number of mammospheres was evaluated by phase contrast microscopy. The box plots indicate medians and 25-75 percentiles and 5-95 percentiles. Differences between groups were analyzed by Wilcoxon signed-rank test. **F.** Phase contrast microscopy pictures of CSC-derived mammospheres from patient 4 with and without treatment with SAL 2 μM at pH 7.4 and pH 6.8. Scale bar: 100 μm. In all charts the * indicates *P* < 0.05.

SAL was shown to be more selective towards breast CSC [40] and it is currently investigated as a promising compound to develop in anti-CSC therapy [52]. We showed that autophagy inhibition by SAL is increased at acidic conditions. We were therefore interested in investigating the effects of pH on cytotoxic activity of SAL in HMLER CD24+ and CD24low cells at pH 7.4 and 6.8. First, we confirmed also in these cells that the cytotoxic activity of CQ is totally inhibited by even mild acidosis (Figure S2B). Thus, we tested the sensitivity of these cells to the dimeric CQ derivative Lys-01 and we observed that CD24low cells are more sensitive to Lys-01 with respect to CD24+ cells (IC_50_ 13.5 ± 1.7 *vs* 30.3 ± 9 μM, *P* = 0.07), indicating that breast CSC are more sensitive to autophagy inhibition (Figure 7A). As expected, the CD24low cells are more sensitive to SAL as compared to CD24+ cells in standard culture conditions at pH 7.4 (*P* < 0.05), in line with their increased susceptibility to autophagy inhibition (Figure 7B and Table 2). Interestingly, the cytotoxic effects of SAL on both cell lines were dramatically amplified in acidic culture conditions (Figure 7B and Table 2), as already observed for HCT116 and Me30966 cells. Importantly, the IC_50_ of SAL for CD24low cells was 10-fold lower than that for CD24+ cells, suggesting an even more selective effect of SAL on these cells at acidic conditions (*P* < 0.05, Table 2). For example, massive vacuolization was observed in CD24low cells exposed to SAL for only 5 hours and cultured at acidic pH while this phenomenon was not seen at pH 7.4 (Figure S2D).

**Table 2:** The ICC_50_ (μM) of SAL in HMLER CD24+ and CD24low cells is reported

	CD24+	CD24low
**pH 7.4**	7.6 ± 1.2	3.1 ± 0.3
**pH 6.8**	1.8 ± 0.3	0.14 ± 0.06

### SAL has pH-dependent effects on inhibition of mammosphere formation

We used HMLER CD24low cells to investigate whether the pH-dependent activity of SAL also affects the CSC property of mammospheres formation. First, we cultured adherent CD24low cells at pH 7.4 and pH 6.8 in presence of increasing concentrations of SAL for 48 hours and investigated the sphere-forming ability of viable single cells after treatment. As shown in Figure 7C, at physiological pH 7.4 culture conditions cells treated with SAL formed significantly less mammospheres as compared to untreated cells (*P* < 0.05). However, this effect was dramatically increased in cells that were treated with SAL under acidic conditions, with < 10% mammospheres formed with SAL 2 μM at pH 6.8 (*P* < 0.05, Figure 7C). In line with this observation, we found that the effects of SAL on reducing the size of mammospheres were also significantly enhanced in acidic conditions (Figure 7D). Similar results were obtained when single cell suspension of CD24low cells were allowed to form mammospheres in the presence or absence of SAL for 7 days (Figure S3A-B).

### Acidic conditions improve the ability of SAL to inhibit mammosphere formation from breast cancer tissue derived stem cells

In order to test the potential relevance of these findings in clinical settings we have analysed the activity of SAL on patient derived breast CSC. Clinical characteristics of tumor patients are provided in Table 3. Briefly, tumor cells were isolated from patient-derived breast cancer tissues and cultured in selective medium as described in Materials and Methods in order to obtain mammospheres (see Figure S3C as example). Mammospheres were then trypsinized and single cells suspensions were cultured at pH 7.4 and pH 6.8 in absence or presence of three SAL concentrations. Newly formed mammospheres were counted after 10 days in culture. First, we could observe that different pH culture conditions did not significantly affect the absolute number of mammospheres formed in untreated cultures (23 ± 8 at pH 7.4 *vs* 17 ± 5 at pH 6.8, *P* = 0.13). As observed for mammospheres from HMLER CD24low cells, SAL inhibited the formation of mammospheres in standard culture conditions in a dose-dependent manner (Figure 7E). Notably, the ability of SAL to inhibit mammosphere formation from patients-derived CSC was dramatically increased in acidic conditions, indicating that SAL has a strong pH-dependent activity also on freshly derived patients CSC (Figure 7F).

**Table 3:** Clinical features of breast carcinoma patients

Patient ID	Tumor type	ER+ (%)	PR+ (%)	Her2+ (%)	KI67+ (%)	NHG
**1**	IDC	100	0	0	21	II
**2**	IDC	95	100	0-1	35	III
**3**	DCIS	NA	NA	NEG	NA	II-III
**4**	IDC	95	0	2	34	III
**5**	IDC	95	45	0	37	III
**6**	IDC	0	0	3	28	III

### Salinomycin accumulates at higher concentrations in cells under acidic conditions

**Figure 8:**
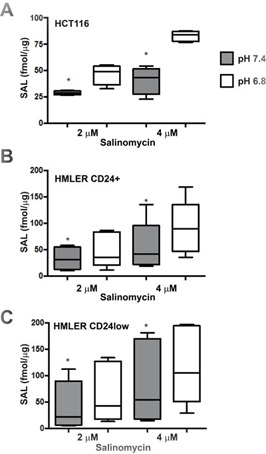
Mass spectrometry data of pH-dependent intracellular SAL accumulation HCT116 **A.**, HMLER CD24+ **B.** and CD24low **C.** cells were plated and the next day exposed to medium buffered at pH 7.4 or pH 6.8 in presence of 2 and 4 μM SAL for 3 hours. The amount of intracellular SAL was quantified by UPLC-MS/MS and expressed as fmol/ μg proteins. Data for HMLER cells represent mean ± SEM of five experimental replicates while data for HCT116 cells are representative of one of two experiments with similar results. Differences between groups were analysed by Wilcoxon signed rank test and the * indicates *P* < 0.05.

A major reason for the lack of anti-autophagic activity of CQ in cancer cells cultured in acidic conditions is the dramatically reduced entry of the drug into cells because of its chemical properties as a weak base with a pKa around 8.1 [36, 53]. SAL contains a carboxylic acid and is a weak acid with a reported experimental pKa of 6.4, which may favor its plasma membrane permeability in acidic conditions. Since SAL showed an improved ability to target cancer cells in acidic conditions we applied UPLC-MS/MS to investigate pH-dependent changes in intracellular SAL accumulation. These experiments were run with two concentrations of SAL (2-4 μM) and in three cell lines (HCT116, HMLER CD24+ and HMLER CD24low cells). The analysis in all cell lines indicated that SAL accumulates in a dose-dependent manner and that the intracellular amount of SAL is significantly higher (up to 50%) in cells cultured in acidic conditions (Figure 8). These results suggest that the intracellular accumulation of SAL may be favored by acidic conditions, which may contribute to the increased activity of SAL in acidic conditions.

## DISCUSSION

Tumor acidosis represents an important pathogenic factor in the progression towards malignant disease and it has been known for many decades to negatively impact on therapeutic efficacy [20, 54]. Autophagy is also known as an important protective mechanism against different forms of anticancer therapies and autophagy inhibition is recognized as a potential tool to improve efficacy of chemotherapy [25]. We and others have reported that melanoma and breast carcinoma cell lines adapt to chronic acidosis by upregulating autophagy, thus providing a rationale for targeting autophagy in acidic tumor regions to prevent malignant progression and improve therapeutic efficacy [34, 35]. The results of phase I/II clinical trials using HCQ as autophagy inhibitor in combination anticancer therapies have indicated potential clinical benefits for a subset of the treated patients but also poor efficacy [33]. Confounding factors for the explanation of these results are the autophagy-independent additional off-target effects of CQ reported recently [55, 56]. We showed that tumor acidosis is one of the factors that may negatively affect the efficacy of CQ *in vivo* [36]. In fact, CQ shows no cytotoxicity and no autophagy inhibition on cancer cells cultured in acidic conditions and in hypoxic tumor regions in a human colon carcinoma xenograft. Such limitations may possibly be overcome by the development of new CQ-derivatives optimized for clinical applications [25, 37]. This scenario prompted us to search for more effective autophagy inhibitors able to target cancer cells during tumor acidosis. The ionophore salinomycin was reported as a compound selectively targeting breast cancer stem cell [40]. The effects of SAL on cancer stem cells have been also reported for other cancer types including glioma and many carcinomas [52, 57–59]. It has been shown that SAL interferes with the maintenance of breast CSC by acting as inhibitor of autophagy and of lysosomal proteolytic function [43]. Here we confirm that SAL at doses 0.2-2 μM potently inhibits the autophagic flux in different tumor cells lines, including osteosarcoma, melanoma, colon carcinoma and breast carcinoma. Importantly, unlike CQ and its derivatives whose activity is to different degrees counteracted by acidosis, the ability of SAL to inhibit autophagic flux is potently increased in conditions of both transient and chronic acidosis. In line with the increased activity as autophagy inhibitor at acidic pH, we found that the cytotoxic activity of SAL was augmented by 5-10 fold in conditions of acidosis when tested on melanoma, colon and breast carcinoma cell lines, with SAL IC_50_ being in the range 100-500 nM in cells cultured at pH 6.8. We observed that the cytotoxic activity of SAL in terms of IC_50_ was similar in cancer cell lines transiently exposed to acidosis and in the same cell lines chronically adapted to acidosis, suggesting that SAL sensitivity may be dictated by medium pH and not by intrinsic properties of the cell lines. Such activity may be related to the pharmacological properties of SAL, which is a weakly acidic, lipophilic compound with a reported pKa of 6.4 due to its carboxylic group [60]. In fact, mass spectrometry analysis of SAL showed an increased intracellular accumulation in cells cultured at pH 6.8 as compared to cells cultured in medium at the physiological pH. However, whether such increased accumulation is alone responsible to explain the stronger activity of SAL in acidic conditions remains to be further investigated. In fact, SAL has an inhibitory effect on oxidative phosphorylation and it is generally considered an inhibitor of mitochondrial function [61, 62], causing a very fast acidification of the mitochondrial matrix. Cell survival under acidosis was reported in post-mitotic cells to be mediated by a homeostatic adaptive response leading to increased mitochondrial function, including maximal respiratory capacity [63]. Interestingly, we observed that acid-adapted HCT116 cells show a higher maximal respiratory capacity as compared to cells cultured at physiological pH (Pellegrini et al, unpublished data) and it is conceivable to hypothesize that SAL-mediated inhibition of mitochondrial function may contribute to increase the sensitivity of cancer cells under acidosis. Moreover, SAL is a potassium ionophore and very likely alters potassium gradients. Interestingly, other potassium ionophores like nigerycin inhibit lysosomal degradation and autophagic flux [64] and there is growing evidence that ions modulate autophagy in cancer [65].

Preclinical pharmacokinetic studies on SAL metabolism performed in mice treated with 5 mg/kg indicated that the C_max_ of plasma SAL concentration was 1.7 μM with a very fast systemic elimination within 5 hours [66]. Interestingly, SAL 5 mg/kg had low systemic toxicity but induced neurotoxic effects which could be reduced using mitochondrial Na^+^/Ca^2+^ exchanger inhibitors without affecting the antitumor effects. In line with this, studies in mice indicated that SAL has reversible dose-dependent adverse effects on the male reproductive system [67]. Therefore, the poor water solubility and its toxicity still represent important limitations for clinical use of SAL, pointing out that SAL derivatives and/or nanoparticles are to be developed to reach higher local concentrations at tumor site [68]. In this context, there is only one report on the treatment of two cancer patients with low dose SAL (250 μg/kg) which showed mild but not long-term side effects [69].

Since SAL has been reported to inhibit CSC in several cancer models we investigated its activity in a model of breast CSC. Two sublines derived from the cell line HMLER and differing in their stem cell properties have been recently described [51]. We first observed that the subline HMLER CD24low, carrying CSC properties has an increased sensitivity to autophagy inhibition mediated by Lys-01 and SAL with respect to the non-CSC subline HMLER CD24+, confirming that autophagy plays a crucial role in maintaining survival and proliferation of CSC [41, 70, 71]. Interestingly, exposure to acidic medium increased the sensitivity of CD24+ cells by 5-fold whereas a 15-fold increase in sensitivity was observed in CD24low cells. In line with this, SAL was more effective at inhibiting autophagic flux in CD24low cells and as low as 200 nM SAL was sufficient to block autophagic flux in CD24low cells cultured in acidic conditions. CD24low cells were described to have CSC features among which the ability to form mammospheres *in vitro* [51]. We found that SAL inhibits mammosphere formation from CD24low cells in standard culture conditions and such inhibition is greatly amplified during acidosis. This indicates that cells with stem-like properties are more susceptible to autophagy inhibition. Moreover it also supports new findings suggesting that inhibition of lysosomal function may specifically target CSC in different cancer types [72, 73]. To further investigate the potential clinical relevance of our observation, we analysed the pH-dependent effects of SAL on CSC derived from patients with breast carcinoma. Breast CSC obtained from cancer patients showed a much greater sensitivity to SAL when cultured in acidic medium.

In conclusion, we identified SAL as a potent inhibitor of the autophagic flux active under tumor acidosis on both cancer cells and CSC. The development of SAL derivatives with lower toxicity for humans will be fundamental to test its efficacy in clinical settings.

## MATERIALS AND METHODS

### Chemical and antibodies

RPMI-1640 (SH30255.01), trypsin (SH40003.12), phosphate-buffered saline (PBS, SH40003.12) and Fetal Bovine Serum (FBS) (SV30160.03) were from HyClone. Sodium Bicarbonate (25080) and RPMI-1640 without NaHCO_3_ (51800) were from Gibco. Bafilomycin A1 (BafA1, B1793), Chloroquine di-phosphate salt (CQ, C6628), salinomycin (SAL, S4526), protease cocktail tablets EDTA-free, phosphatase inhibitors (P5726, P0044), insulin (I9278) and bovine serum albumin (BSA, A7906) were from Sigma. Recombinant human epidermal growth factor (EGF) was purchased from Invitrogen (PHG6045). The Lys-01 was synthetized by OncoTargeting AB (Sweden) and dissolved in DMSO. Protein assay dye reagent concentrate (500-0006), protein assay standard I (5000-0007) and dry milk (170-6404) were from Bio-Rad. The following antibodies were used: LC3B (Cell Signaling Technology, 2775) and β-actin (Sigma, A5441). HRP-conjugated anti-rabbit (NA934V) and anti-mouse (NXA931) antibodies, ECL system (RPN2106) and PVDF membranes (RPN303F) were from GE Healthcare.

### Cell culture

Me30966, HCT116 and HOS-GFP-LC3 cell lines were cultured in RPMI-1640 medium supplemented with 10% FBS and antibiotics. The HOS cell line stably expressing the GFP-LC3 plasmid (HOS-GFP-LC3) was a kind gift from Gerry McInerney (MTC, Karolinska Institute, Stockholm). The cell lines HMLER CD24+ and HMLER CD24low were kindly provided by Dr. Anne-Pierre Morel (Centre Leon Berard, Lyon, France). These cells were grown in DMEM-F12 medium supplemented with 10% FBS, antibiotics, insulin (10 μg/ml), hydrocortisone (0.5 μg/ml), EGF (10 ng/ml) and puromycin (0.5 μg/ml). All cells lines were grown at 37°C in presence of 5% CO_2_. The low pH adapted HCT-116 (HCT116_pH6.8_) and Me30966 (Me30966_pH6.8_) cell lines were obtained by growing the parental cells in RPMI-1640 medium buffered at pH 6.8 for three months as previously described [36]. The different pH in media was buffered by adding different concentration of NaHCO_3_ and letting the media equilibrate overnight in the incubator at 5% CO_2_. Actual pH in media was measured before, during and after each experiment. Cell lines were tested using the LGC Standards Cell Line Authentication service.

### High-content fluorescence microscopy

HOS-GFP-LC3 cells (7000 cells/well) were plated into a 96 well plate (Cell carrier, 6005550) and incubated at 37°C and 5% CO_2_ overnight. The medium was removed at the following day and replaced with medium at pH 7.4 or pH 6.8. SAL was added for 6 h and BafA1 for the last 2 h of the incubation. The medium was removed and the cells were incubated with 4% paraformaldehyde and 5μg/ml Hoechst 33342 (Thermo Fischer, H3570) in the dark at RT for 20 minutes. The paraformaldehyde was removed and the cells were washed three times using PBS and kept in PBS during acquisition. Images were captured using an ImageXpress scanner. The software MetaXpress was used to analyse the pictures defining GFP-positive dots of 0.5-8 μm as GFP-LC3-positive vesicles. ImageJ was used to compose the pictures of the different wavelenghts.

### Multicellular spheroids

HCT116 cells (10000 cells in 370 μL) were added to each well of ultra low 96-well plates and the plates were inverted to allow the cells to sediment for 24 hr. After flipping the plates, newly formed MCS were allowed to sediment and were incubated for 5 days before treatment with compounds. At this stage MCS had a diameter of about 600 μm.

### Immunohistochemistry

For experiments in which MCS were stained for LC3 expression by immunoistochemistry, MCS were treated with BafA1 (50 nM), CQ (50 μM), Lys-01 (50 μM) and SAL (2 μM) for 24 hours before processing samples. MCS from each treatment group (*n* = 24) were fixed in 2% buffered formalin, dehydrated, embedded in paraffin and 10 μm sections were obtained. The sections were deparaffinized with xylene, rehydrated and microwaved. Sections were stained with rabbit anti-human LC3B (Cell Signaling Technology, 2775) and visualized by avidin-biotin-peroxidase complex technique (Vector Laboratories, Burlingame, CA, USA). Counterstaining was done using Mayer's haematoxylin.

The analysis of LC3 expression was performed by two independent operators using the software Aperio ImageScope and the algorithm positive pixel count v9, as described previously [36]. Since the basal level of LC3 expression in untreated MCS is weak, treatment with any of the autophagy inhibitors tested will block turnover of LC3-II and induce increased LC3 expression. LC3 expression was calculated as number of positive pixels (including positive and strong positive pixels). The peripheral area (roughly 10 cellular layers) and the central area of each MCS, respectively characterising proliferating/normoxic cells and quiescent/hypoxic cells [74] were analysed and LC3 expression was normalised to total area and expressed as percentage.

### Mammosphere formation

After treatment with SAL for 48 hours, HMLER CD24low cells were collected and resuspended in DMEM/F12 medium supplemented with B27 (1:50, Invitrogen, 17504044), 20 ng/ml EGF, 0.4% BSA, 0.5 μg/ml hydrocortisone, 4 μg/ml insulin, as previously described [43]. For each treatment group, cells were plated at 1 cell/well/150 μl in one ultralow attachment 96-wells plate (Costar, 7007), one full plate per condition. After 5-7 days, the number and size of mammospheres formed in each plate were counted and analysed. In some experiments, HMLER CD24low cells were directly cultured as single cell suspension in the presence of SAL for 7 days and the number of mammospheres formed was evaluated.

### Clinical samples

Fresh biopsies and scrapings from patients with primary breast cancer (Table 3) were obtained from the Karolinska Hospital and immediately processed. Research on the tumor samples was approved by the local biobank at Karolinska University Hospital and the Regional Ethics Board in Stockholm, Sweden. Fresh biopsies and/or tumor cells were directly generated from surgically resected breast cancers at Karolinska University Hospital as previously described [75]. Tumor cells were immediately transferred into the selective medium DMEM/F12-GlutaMAX supplied with 10 ng/ml b-FGF, 20 ng/ml EGF, 5 μg/ml insulin and 0.4% BSA for the initiation of mammospheres. In general, scrapings were washed twice in PBS, the final cell pellets were re-suspended in the selective medium and stable mammospheres can be observed after 3 days (Figure S3C). Sensitivity to SAL was tested on single cell suspensions obtained from dissociated mammospheres and cultured in DMEM-F12 buffered at pH 7.4 or 6.8 at 1000 viable cells per well in 48-well plates (Corning Costar, MA) to form mammospheres. Plates were incubated for 10 days and the number of newly formed mammospheres was documented.

### Studies on autophagic flux

Mild saponin extraction of cytosolic GFP-LC3-I allows quantification of autophagosome-associated GFP-LC3-II by flow cytometry in HOS-GFP-LC3 cells [48]. HOS-GFP-LC3 cells (100,000 cells) were plated into 20 cm^2^ dishes. The next day medium was replaced with medium at pH 7.4 or 6.8 for 24 hours. SAL was added for 6 hours and BafA1 (50 nM) was added during the last 2 hours incubation. Cells were collected by trypsinization and treated with 0.05% saponin (Biochemika, 47036) in PBS for 10 minutes at RT. After wash in PBS the cells were collected and immediately analysed by a FACSCalibur instrument using Cellquest software (Becton Dickinson). GFP fluorescence was collected from 10,000 cells/sample.

Analysis of the autophagic flux by Western blot was done on different cell lines. Experiments with parental cell lines were performed by plating cells in standard RPMI buffered at pH 7.4. The next day the medium was replaced with media buffered at the desired pH (7.4, 6.8 and 6.5). Low pH-adapted cell lines were plated in medium buffered at pH 6.8 and let adhere overnight. SAL was added at specified concentrations for 6 hours and 100 nM BafA1 was added during the last 2 hours incubation. Cells were then collected for further analysis by flow cytometry or Western blotting.

Analysis of autophagy in HMLER CD24+ and CD24low cells was performed using an mRFP-GFP-LC3 reporter. Adherent cells were transfected with the mRFP-GFP-LC3 plasmid by using lipofectamine 3000. The next day, medium was replaced with fresh medium buffered at the desired pH. After overnight incubation, SAL was added for 6 hours and EBSS-treated cells were used as controls. Cells were fixed using 2% paraformaldehyde and observed by laser scanning fluorescence microscopy. Autophagic flux was determined by quantification of the number of autophagosomes (yellow dots) and autolysosomes (red dots) per cell, counting at least 15 cells per condition.

### Western blotting

Cells were washed with PBS on ice and collected by scraping in cold PBS. The cell pellet was lysed in RIPA buffer (150 mM NaCl, 50 mM Tris pH 7.4, 1% Nonidet P-40, 0.1% SDS and 0.5% sodium deoxycholate) in presence of protease and phosphatase inhibitors. The protein concentration was determined by Biorad Protein Assay (Biorad Laboratories) and equal amount of proteins (20 μg) was loaded on pre-casted acrylamide gels (4-12% SDS-PAGE, NuPage). The proteins were transferred from the gel to PVDF membrane for 2 hours at 4°C. Red Ponceau staining of the membranes verified the proper loading and transfer. Membranes were blocked in 5% blotting grade dry milk in TBS with 0.1% Tween (TBS-T) for 1 hour at room temperature and then incubated with primary antibodies diluted in 5% BSA in TBS-T overnight at 4°C. The next day membranes were washed and incubated for 1 hour at room temperature with the appropriate HRP-conjugated secondary antibody and the binding revealed by the ECL system.

### Cell viability assay

Cells were plated into 96-wells plates and the next day treated with different concentrations of the specific compounds (SAL, CQ and Lys-01). Forty-eight hours after treatment cell viability was evaluated by using the acid phosphatase assay [76].

### Clonogenic assay

HCT116 cell lines were plated as 300 cells/well in 6-well plates and treated with SAL for 8 or 48 hours, following culture for 6 days in drug-free media. Colonies were stained with Giemsa.

Clonogenic cell survival was also assessed on HCT116 MCS treated for three days with different compounds. After treatment, the MCS were collected, washed in PBS and single cell suspension was obtained after incubation with Accutase (Biolegend, 423201). Equal volumes of cells for each treatment condition were plated in triplicate in 6-well plates for 6 days, followed by staining the colonies with Giemsa. The software ImageJ was used to perform colony counting.

### UPLC-MS/MS analysis of salinomycin intracellular content

The intracellular content of SAL was examined using a method commonly used for assessing drug uptake/accumulation [77]. In brief, HCT116, HMLER CD24+ and CD24low cells were plated in 24-wells plates overnight and the next day medium was replaced with fresh medium at pH 7.4 and pH 6.8. Immediately after, SAL (2-4 μM) was added to the cells for 3 hours. After the 3h incubation cells were washed once with PBS at room temperature and then acetonitrile was added and let evaporate. The intracellular accumulation of SAL was quantified by ultra-high performance liquid chromatography-tandem mass spectrometry (UPLC-MS/MS). The compounds accumulated were extracted with 200 μl acetonitrile/water (60/40) spiked with 50 nM warfarin as internal standard. The samples were centrifuged at 2465g and 4°C for 20 minutes. Accumulation of SAL was determined using a Waters Xevo triple quadrupole mass spectrometer with electrospray ionization coupled to an Acquity UPLC system (Waters, Milford, MA, USA). Compounds were separated with a 2 min elution at a flow rate of 0.5 ml/min in a Waters BEH C18 column, 2.1 × 50 mm (1.7 μm) at 60°C. Mobile phase consisted of 5% acetonitrile and 0.1% formic acid in water (solvent A), and 0.1% formic acid in acetonitrile (solvent B). The chromatographic run started at 20% solvent B and increased linearly to 99% from 0.5 to 1.2 min, followed by a hold until 1.6 min, and a return to initial conditions at 1.7 min. SAL was detected by monitoring the mass transition 749>241 at a cone voltage of 48V and a collision energy of 33V. SAL eluted at 1.75 min. For all cell lines the total protein content was measured in representative wells using the BCA Protein Assay Reagent Kit (Pierce Biotechnology, Rockford, IL, USA) according to manufacturer's instructions. The obtained protein contents were used for normalization between experiments and cell lines.

### Statistical analysis

All data were obtained from at least three experimental replicates and presented as mean ± SEM, if not otherwise indicated. Data analysis was performed using Graphpad Prism 6.0 (GraphPad Software, CA). Differences between groups were analysed with parametric or non-parametric tests according to the distribution of the values. The specific test used is indicated in the legend to figures. The significance level was set as *P* < 0.05.

## SUPPLEMENTARY MATERIAL FIGURES


