# Supplementary material for: Tumor acidosis enhances cytotoxic effects and autophagy inhibition by salinomycin on cancer cell lines and cancer stem cells
Source: Oncotarget. 2016 May 27;7(24):35703–23. doi: 10.18632/oncotarget.9601 (PMC5094956; doi:10.18632/oncotarget.9601)
Supplement: Supplementary file 1 [file oncotarget-07-35703-s001.pdf]

Tumor acidosis enhances cytotoxic effects and autophagy inhibition by salinomycin on cancer cell lines and cancer stem cells

Supplementary Material

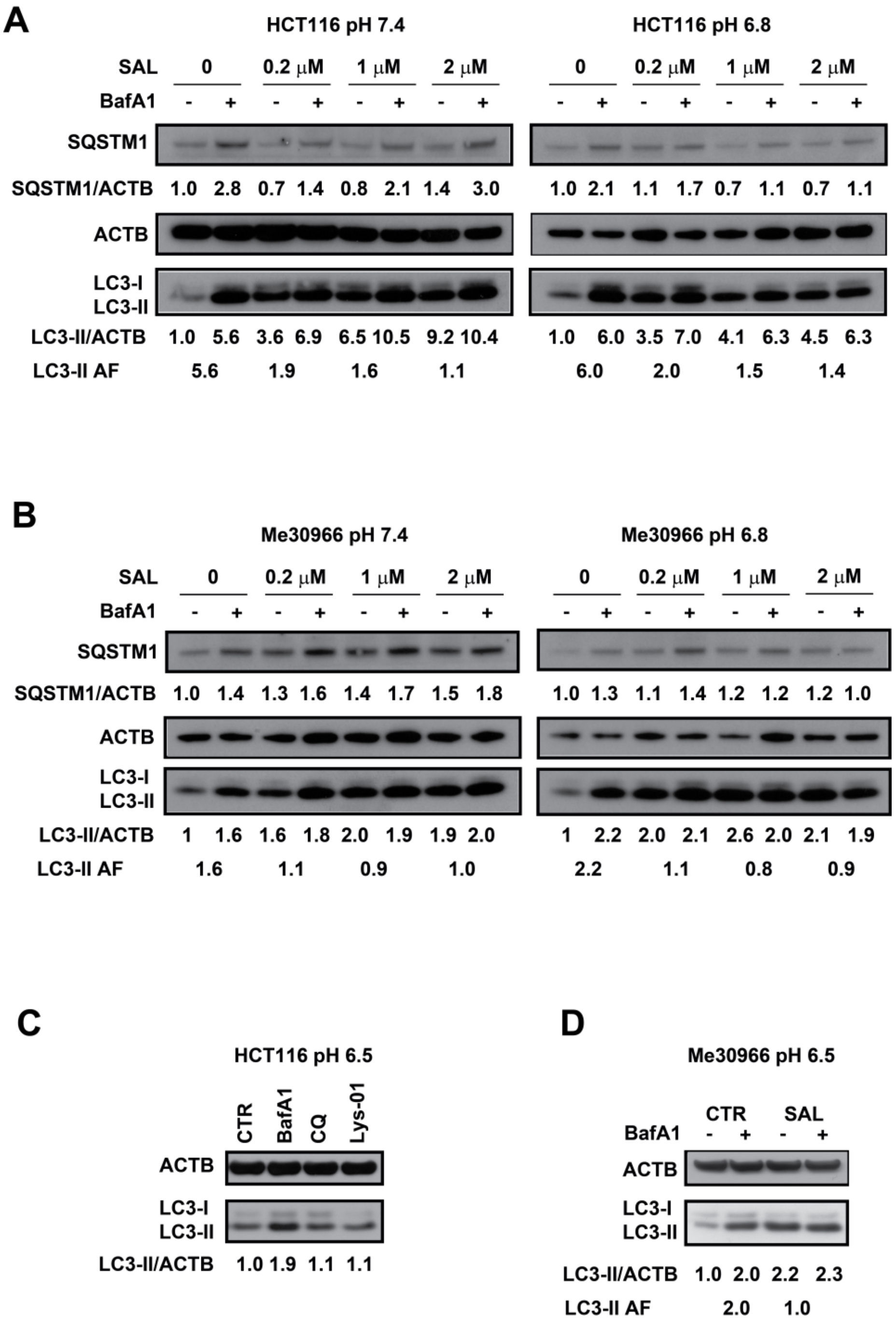

Figure S1. Autophagy inhibition by SAL in different cell lines. SAL has a dose-dependent effect on blocking autophagic flux in parental and low pH-adapted HCT116 (A) and Me30966 (B) cells. (C) HCT116 cells transiently cultured at pH 6.5 maintain the autophagic flux which is inhibited by BafA1 but not by CQ or its derivative Lys-01. (D) SAL is able to completely block the autophagic flux of Me30966 cells transiently exposed to medium at pH 6.5. Experiments in this figure have been repeated three times.

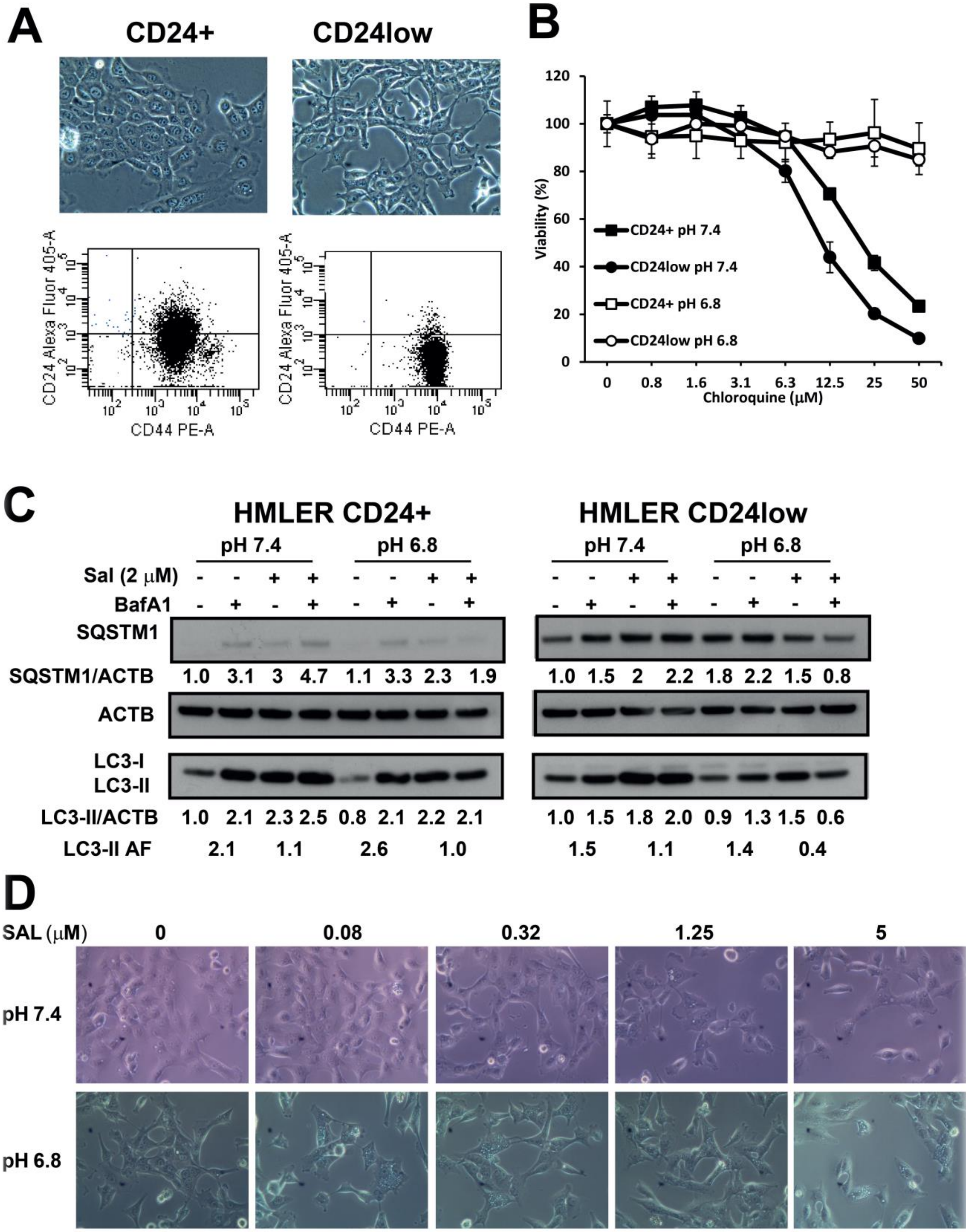
A
CD24+
CD24low
CD24 Alexa Fluor 405-A
CD44 PE-A
B
Viability (%)
Chloroquine (μM)
CD24+ pH 7.4
CD24low pH 7.4
CD24+ pH 6.8
CD24low pH 6.8
C
HMLER CD24+
HMLER CD24low
pH 7.4
pH 6.8
Sal (2 μM)
BafA1
SQSTM1
SQSTM1/ACTB
1.0 3.1 3 4.7 1.1 3.3 2.3 1.9
1.0 1.5 2 2.2 1.8 2.2 1.5 0.8
ACTB
LC3-I
LC3-II
LC3-II/ACTB
1.0 2.1 2.3 2.5 0.8 2.1 2.2 2.1
1.0 1.5 1.8 2.0 0.9 1.3 1.5 0.6
LC3-II AF
2.1 1.1 2.6 1.0
1.5 1.1 1.4 0.4
D
SAL (μM)
0
0.08
0.32
1.25
5
pH 7.4
pH 6.8

Figure S2. Sensitivity of HMLER cells to autophagy inhibition. (A) Phase contrast microscopy and FACS analysis for CD24/CD44 expression of the non-CSC cells HMLER CD24+ and the CSC cells CD24low are shown. (B) HMLER cells CD24+ and CD24low are sensitive to CQ-mediated cytotoxicty at standard pH culture conditions but completely insensitive to CQ in acidic conditions. (C) HMLER CD24+ and CD24low cells were plated and cultured with medium buffered at pH 7.4 or pH 6.8 overnight. SAL (2 μM) was added for 6 hours, with or without BafA1 (100 nM) during the last 2 hours incubation. (D) Representative phase contrast microscopy pictures of HMLER CD24low cells cultured at pH 7.4 and pH 6.8 and treated for 5 hours with different concentrations of SAL. Optical microscopy shows that 5 hours treatment with SAL as low as 0.08 μM induces a dramatic increase in vacuolization in CD24low cultured at pH 6.8 while the effects at pH 7.4 are barely observed.

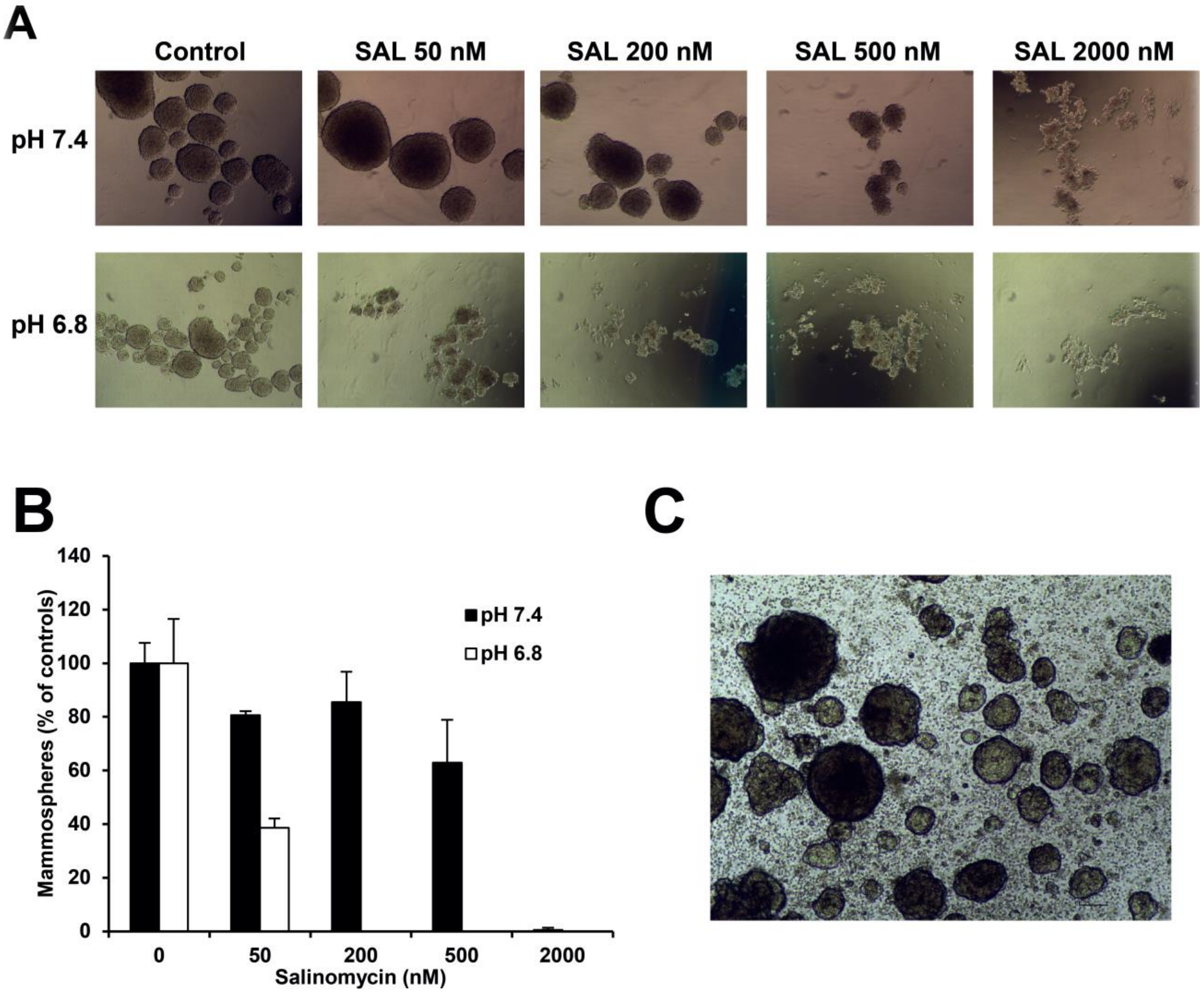

Figure S3. Effects of SAL on mammospheres. HMLER CD24low cells were cultured in stem cells medium buffered at pH 7.4 or pH 6.8 as single cells suspensions in the presence of various concentrations of SAL. After 7 days optical microscopy pictures were taken (A) and the number of mammospheres was analysed (B). This experiment was performed three times. (C) Phase contrast microscopy of mammospheres obtained from breast carcinoma derived mammospheres before dissociation and treatment with SAL. Scale bar: 100 μm.
